# Proteome-Wide Profiling of the MCF10AT Breast Cancer Progression Model

**DOI:** 10.1371/journal.pone.0011030

**Published:** 2010-06-09

**Authors:** Lee Yee Choong, Simin Lim, Poh Kuan Chong, Chow Yin Wong, Nilesh Shah, Yoon Pin Lim

**Affiliations:** 1 Cancer Science Institute of Singapore, National University of Singapore, Singapore, Singapore; 2 Department of General Surgery, Singapore General Hospital, Singapore, Singapore; 3 Department of Biological Sciences, Faculty of Science, National University of Singapore, Singapore, Singapore; 4 Bioinformatics Institute, Agency for Science, Technology and Research, Singapore, Singapore; Health Canada, Canada

## Abstract

**Background:**

Mapping the expression changes during breast cancer development should facilitate basic and translational research that will eventually improve our understanding and clinical management of cancer. However, most studies in this area are challenged by genetic and environmental heterogeneities associated with cancer.

**Methodology/Principal Findings:**

We conducted proteomics of the MCF10AT breast cancer model, which comprises of 4 isogenic xenograft-derived human cell lines that mimic different stages of breast cancer progression, using iTRAQ-based tandem mass spectrometry. Of more than 1200 proteins detected, 98 proteins representing at least 20 molecular function groups including kinases, proteases, adhesion, calcium binding and cytoskeletal proteins were found to display significant expression changes across the MCF10AT model. The number of proteins that showed different expression levels increased as disease progressed from AT1k pre-neoplastic cells to low grade CA1h cancer cells and high grade cancer cells. Bioinformatics revealed that MCF10AT model of breast cancer progression is associated with a major re-programming in metabolism, one of the first identified biochemical hallmarks of tumor cells (the “Warburg effect”). Aberrant expression of 3 novel breast cancer-associated proteins namely AK1, ATOX1 and HIST1H2BM were subsequently validated via immunoblotting of the MCF10AT model and immunohistochemistry of progressive clinical breast cancer lesions.

**Conclusion/Significance:**

The information generated by this study should serve as a useful reference for future basic and translational cancer research. Dysregulation of ATOX1, AK1 and HIST1HB2M could be detected as early as the pre-neoplastic stage. The findings have implications on early detection and stratification of patients for adjuvant therapy.

## Introduction

Cancer is the result of a multi-step process involving initiation, propagation and maintenance of cancer cells. Each individual step and its transition to the next require accumulation of aberrations associated with an intricate network of genes. A better understanding of the molecular etiology and therefore a more effective management of breast cancer requires a systems biology approach as opposed to the classical one gene/one pathway approach. The use of various genomics, proteomics technology platforms and biological systems has provided much insight into these areas [1]. However, understanding disease progression is not without challenges. For example, the study of clinical samples is complicated by cellular, genetic, environmental and treatment heterogeneities. On the other hand, it is difficult to ascertain whether changes observed were indeed associated with cancer or due to variations in genetic backgrounds when using non-isogenic *in vitro* cell models.

Isogenic cell lines are advantageous and have been used widely for studying molecular events during disease development and drug resistance. First developed in Fred Miller's laboratory, the MCF10AT model comprises at least four isogenic cell lines MCF10A1, MCF10AT1K.cl2, MCF10CA1h and MCF10CA1a.cl1 that represent normal, premalignant epithelium, low grade and high grade lesions, respectively [2], [3]. MCF10A1 cells are not tumorigenic in nude mice while MCF10AT1K.cl2 cells could form simple ducts that progress into benign hyperplasia and occasionally carcinoma. MCF10CA1h formed largely well differentiated carcinoma while MCF10CA1a.cl1 produced poorly differentiated carcinoma and could metastasize to the lung in tail vein injection assay. The MCF10AT model has several salient features of proliferative breast disease in humans including the histological spectrum of lesions and heterogeneity within a single host [4]. This model has proven to be useful for cancer related studies including cytogenetics, DNA damage, apoptosis and TGF-β signaling [5], [6], [7], [8].

Recently, mRNA expression profiling and copy number variation of the MCF10AT model were conducted [9], [10]. However, the mRNA level, copy number and protein level do not necessarily correlate well. Since proteins are the workhorses of the cells and >90% of all drug targets are protein in nature, we proposed that proteomic analysis of the MCF10AT model is complementary and informative. Although current proteome-wide technologies could only detect a few thousand proteins at best, making it not a truly systems biology tool, it nevertheless can generate a useful reference database for future basic and translational cancer research.

Several technologies that emerged at the turn of the millennium are available for shot-gun protein expression profiling. They include isotope-coded affinity tag (ICAT), isobaric tags for relative and absolute quantification (iTRAQ) and stable isotope labeling with amino acids in cell culture (SILAC) and have been reviewed elsewhere [11], [12]. Among them iTRAQ is a powerful tool in which up to eight samples can be relatively quantified in one experiment thereby reducing inconsistency between analyses [13]. In this study, iTRAQ was used to generate a list of proteins that display expression changes during breast cancer progression as modeled by the MCF10AT system. Subsequent studies validated the aberrant expressions of some candidate proteins in the progression of clinical breast cancers.

## Materials and Methods

### Reagents

Anti-mouse IgG (whole molecule), anti-rabbit IgG (whole molecule) and Cy3-conjugated anti-Vimentin antibodies were purchased from Sigma Chemical Co. (St. Louis, MO). Anti-ATOX1 mouse monoclonal antibodies, anti-VCP and anti-Histone H2B antibodies were from Abcam (Cambridge, UK). Anti-AK1, anti-LGALS3 rabbit polyclonal antibodies and HRP-conjugated anti-actin mouse monoclonal antibodies were from Santa Cruz Biotechnology Inc. (CA, USA).

### Cell lines, sample preparation and immunoblotting

Xenograft-derived breast cancer cell lines (MCF10A1, MCF10AT1KCl.2, MCF10CA1h and MCF10CA1aCl.1) were obtained from Dr Fred Miller at the Barbara Ann Karmanos Cancer Institute (Detroit, MI). This biological system has been extensively reviewed [14], [15], [16] and cell lines are cultured as described in many studies [5], [6], [7], [8], [9], [10]. Cells were incubated at 37°C in a humidified atmosphere containing 5% CO_2_ until 95% confluence when the medium was replaced with serum- and additives-free media overnight to study the basal protein expression. Cells were rinsed with ice-cold PBS and lysed on ice for protein extraction using iTRAQ lysis buffer: 0.2% IGEPAL, 0.2% Triton X, 0.2% w/v CHAPS, 75 mM NaCl, 1 mM EDTA, 50 mM sodium fluoride, protease inhibitor and 1 mM sodium orthovanadate in PBS. Protein lysates were then clarified by centrifugation at 4°C at 14,000 rpm for 10 min. Total protein was determined using the bicinchoninic acid assay (BCA) kit (Pierce Biotechnology). Immunoblotting was performed as per previous reports [17], [18].

### Isobaric peptide labeling and nanoLC-MS/MS analysis

A total of 100 µg of protein from each sample was reduced, alkylated, digested and labeled with iTRAQ reagents according to the manufacturer's protocol (Applied Biosystems, Framingham, MA, USA). Specifically, proteins from MCF10A1, MCF10AT1k.cl2, MCF10CA1h and MCF10CA1a.cl1cells were labeled with 114, 115, 116 and 117 tags respectively. The dried, labeled peptides were then constituted and subject to ion exchange chromatography as previously described[19]. A total of 23 fractions were collected and these fractions were dried in vacuum concentrator, and stored at −20°C prior to mass spectrometric analysis. Mass spectrometry was performed using a QStar XL Hybrid ESI Quadrupole time-of-flight tandem mass spectrometer, electrospray ionization quadruple-time of flight tandem-mass spectrometry (ESI-qQ-TOF-MS/MS) (Applied Biosystems, Framingham, MA, USA; MDS-Sciex, Concord, Ontario, Canada) coupled with an online nanoflow liquid chromatograph (Agilent 1100 system from Agilent, Santa Clara, USA). Protein identification and quantification for iTRAQ samples were carried out using ProteinPilot™ software (version 2.0; Applied Biosystems, MDS-Sciex). Only proteins identified with at least 95% confidence i.e., p≤0.05 were reported. Other details were as previously described [19]. To estimate the rate of false positive in the dataset obtained, we employed a database search strategy against a concatenated pseudo-reverse database [20]. This database was created in-house, consisting 16,602 human sequences and their pseudo reverse sequences. Here we defined FDR as the percentage of decoy proteins identified against the total protein identification.

### Immunofluorescence

Cells were grown on Menzel microscope coverslip till about 50–60% confluence. The coverslip was rinsed in three changes of PBS, pH 7.4 and fixed with 3.7% paraformaldehyde, permeabilized in PBS containing 0.5% Triton X. Following blocking with 1% BSA, cells were incubated with Cy3-conjugated anti-Vimentin mouse monoclonal antibody (1∶1000 dilution) at 37°C for 1 h. After washing 3 times with PBST (PBS+0.1% Tween 20) for 3 min each, cells were counterstained with DAPI for 1 minute. Cells were then mounted on glass slide with prolong anti-fade reagent. Analyses were done using Zeiss dark field fluorescence microscope with barrier filter 50, interference filter KP-500, and a 100-W quartz Halogen lamp light source was used.

### Clinical samples and Immunohistochemistry

Frozen matched malignant and adjacent normal breast tissues were requested from the Tissue Repositories (TRs) of NCCS and NUH (retrospective accrual) following approvals from Institutional Review Boards from the National Cancer Centre of Singapore (NCCS), National University Hospital (NUH) and the National University of Singapore. They are mainly primary tumors and they were stored in liquid nitrogen before use. We have no information on the treatment history of these samples. Frozen tissues were prepared for IHC by first fixing them in 10% neutral buffered formalin (Sigma) for 16 h at 4°C, subject to ThermoShandon tissue processor and embedded in paraffin. Paraffin sections were warmed in a 60°C oven and dewaxed in three changes of xylene and passaged through graded ethanol (100%, 95%, 70%) before a final wash in ddH_2_0. For ATOX1, antigen retrieval was performed via pressure cooking at 120°C for 5 min in Tris-EDTA buffer, pH 9.0. For HIST1H2BM and AK1, antigen retrieval was performed using the Target Retrieval Solution (Dakocytomation, Denmark) at 95°C for 40 min. After quenching of endogenous peroxidase activity with 3% H_2_0_2_ for 10 min and blocking with 5% BSA for 30 min, sections were incubated at 4°C for overnight with antibodies against the primary antibodies at 1∶25 to 1∶100 dilutions. Detection was achieved with the Envision+/HRP system (Dakocytomation, Denmark). All slides were counterstained with Gill's hematoxylin for 1 min, dehydrated and mounted for light microscopic evaluation.

### IHC scoring and statistical analyses

Interpretation of H&E sections and analysis/scoring of IHC data were all done by certified pathologists (NS). Expression of proteins revealed by IHC was first scored (3+ = 3, 2+ = 2, 1+ = 1, 0 = negative; NA = scores not available due to lack of ductal components). In cases where different degrees of staining were observed within the same section, average values were taken e.g. 2+/1+ = 1.5. Where staining was observed only for certain areas within the section, the entire section was surveyed to estimate the percentage of area with positive signal. Hence for scores with percentage within parenthesis, e.g. 2+ (50%), the final score was obtained by the multiplying 2×0.5 = 1.0.

## Results

### Detection and relative quantification of proteins across the MCF10AT model of breast cancer progression

**Figure 1:**
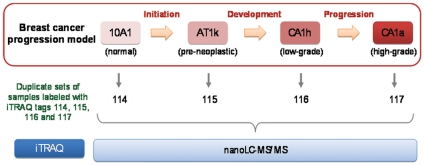
Overview of iTRAQ-based protein expression profiling of MCF10AT breast cancer progression model. Biological duplicates were prepared. Lysates from A1, 1k, 1h and 1a cells were labeled with 114, 115, 116 and 117 iTRAQ isotopic labels, respectively, and analyzed with nanospray-ESI tandem mass spectrometry.

The cell lines used in this study MCF10A1, MCF10AT1K.cl2, MCF10CA1h and MCF10CA1a.cl1 are abbreviated as A1, 1k, 1h and 1a, respectively. In our laboratory, the tumors obtained by subcutaneous injection of 1k, 1h, and 1a cancer cells into nude mice grew at different rates and were of increasing grades (degree of differentiation) as assessed by our pathologist (NS) validating that the model was indeed reflective of disease progression as originally reported by Miller's group [21]. The experimental design is shown in Figure 1. Two biological preparations were made and analyzed independently to achieve greater accuracy. Following analysis with ProteinPilot™ software, >1000 proteins were detected with 95% confidence and the relative expression levels of proteins determined as iTRAQ ratios.

To filter out proteins that display high confidence expression changes across the MCF10AT model, we performed a 2-step data processing. First, we implemented a 1.3 fold cut off on the iTRAQ ratios to segregate proteins into those that were up or down regulated. This cut-off value was applied since several iTRAQ studies conducted in our laboratory revealed that the technical variation was consistently below 30%. Therefore, the upper and lower range worked out to be 1.3 (1×1.3) and 0.77 (1/1.3), respectively. Proteins with expression ratios below the lower range were considered to be under-expressed while those above the higher range were considered over-expressed. Second, only iTRAQ ratios that fulfilled the 1.3 fold criteria AND are statistically significant ratios are highlighted in red (for up-regulation) and green (for down-regulation). This resulted in Supplementary Table S1, which shows 98 proteins with significant difference in expression level across one or more stages of breast cancer progression as modeled by the MCF10AT system. The protein detection (including group reporting and% coverage) and other relative quantification data including p-value, error factor (EF) and% coverage from iTRAQ/ESI-based LC-MS/MS analyses are provided as Supplementary Table S2. The false discovery rate (FDR) for iTRAQ/ESI-based data worked out to be 1.2%.

### Characteristics of the proteomic changes across the MCF10AT model

**Figure 2:**
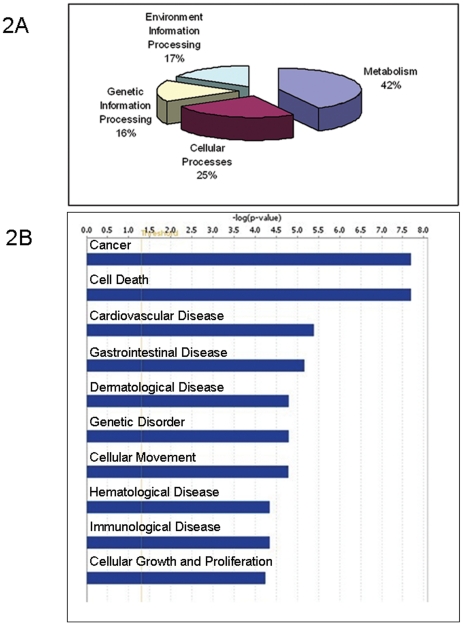
Functional characterization of proteins detected to display aberrant expression across the MCF10AT model of breast cancer progression. (**A**) Proteins were organized via KO (KEGG Orthology) using KEGG pathway database into major processes such as Metabolism (carbohydrate, lipid, amino acid etc.); Genetic Information Processing (transcription, translation, replication, repair etc.); Environmental Information Processing (membrane transport, signal transduction etc.); and Cellular Processes (cell growth, death, motility etc.). (**B**) Gene list was imported and analyzed by the Core Analysis Module in Ingenuity Pathway Analysis software to statistically determine the functions/pathways most strongly associated with the gene list.

From Supplementary Table S1, we observed that the number of proteins that showed different expression levels increased as disease progressed from AT1k pre-neoplastic cells to low grade CA1h cancer cells and high grade cancer cells. This reflects an immense degree of aberrations when cancer transit from low to high grade cancers. Since this study represents the first proteome-wide analysis of the MCF10AT model of breast cancer progression, it was necessary to understand the nature of the proteins involved in the process. To this end, 98 proteins were classified via KO (KEGG Orthology) using KEGG pathway database [22], [23]. Figure 2A shows that cancer progression is associated with a major re-programming in metabolism (42%), one of the first identified biochemical hallmarks of tumor cells (the “Warburg effect”). To characterize the functions associated with proteins detected, the gene list was uploaded into Ingenuity Pathways Analysis (IPA) software server and analyzed using the Core Analysis module as per manufacturer's instructions [24]. As seen in Figure 2B, the top two functions that the gene list is most significantly related to is cancer and cell death. This suggests that the list of genes detected in this study is mostly regulators/effectors of cancer cell growth and proliferation.

### Major classes of proteins implicated in the MCF10AT model of breast cancer development

To organize the data in Supplementary Table S1, proteins were classified into their respective molecular functions using PANTHER [25]. Supplementary Table S3 shows the classification of 98 proteins into the various molecular functions. A few molecular function classes associated with the salient phenotypes of cancer were selected for discussion.

#### (A) Calcium-binding proteins

Calcium is an important secondary messenger and proteins that regulate calcium fluxes or whose functions are regulated by calcium are important regulators of cancer cell biology [26]. The S100 family of proteins has at least 25 members, most containing 2 EF-hand calcium binding motifs. They have been shown to play diverse roles ranging from regulation of calcium release, microtubule assembly and signaling pathways [27]. Close to ten S100 calcium-binding proteins were detected in our dataset. Aberrant expressions of these proteins have been implicated in the pathogenesis of many human cancers with some members (e.g. A100A4 and A8) being associated with cancer invasiveness and metastasis [28], [29]. Consistent with the DNA microarray analysis of the MCF10AT system [9], S100A8 and S100A9 were found to be down-regulated in our study. In addition, we have identified up-regulation of several other S100 proteins including A2, A11, A13, A14 and A16, abnormal expressions of which have been associated with bladder and esophageal carcinomas [30], [31].

#### (B) Cytoskeletal proteins

**Figure 3:**
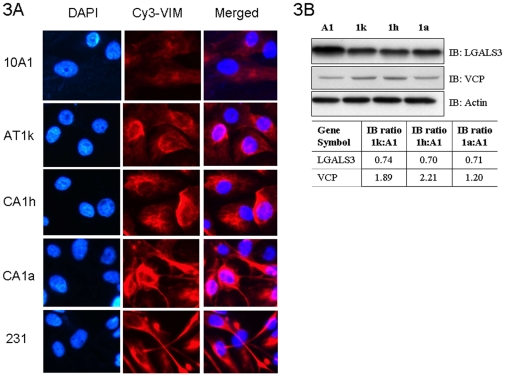
Expression profiles of Vimentin, LGALS3 and VCP across the MCF10AT model. (**A**) Immunofluorescence of Vimentin in MCF10AT model using Cy3-conjugated anti-Vimentin antibodies. (**B**) **Top panels -** Immunoblotting of LGALS3 and VCP. Cells were processed as per iTRAQ experiments. The lysates were immunoblotted with the protein-specific antibodies to reveal the relative expression levels across the 4 MCF10AT cell lines. Actin was included as a loading control. **Bottom panel** -Densitometry readings for the signals corresponding to the respective protein bands were obtained and expressed as a ratio using the signal from A1 normal cells as the denominator

Transition of cancer cells from non-invasive to invasive phenotype is usually accompanied by epithelial-mesenchymal transition (EMT). One of the traits associated with EMT is enhanced migratory capacity. Interestingly, Vimentin, an intermediate filament cytoskeletal protein and a marker of EMT was shown by this study to be significantly up-regulated in CA1a high grade cancer cells. As the Vimentin antibodies we purchased were not good for immunoblotting, we performed immunofluorescence of Vimentin (VIM) across the MCF10AT cells lines. Highly invasive MDA-MB-231 (231) cell line was included as a control. Although IF is only semi-quantitative, it clearly revealed very strong Vimentin expression in CA1a high grade cancer cells compared to 10A1 normal mammary epithelial cells (Figure 3A). Like MDA-MD-231, CA1a cells displayed a more fibroblastic morphology that is characteristic of highly migratory cells, compared to the rest of the cell lines. Intermediate filaments are important to cell migration, invasion and metastasis [32]. They extend from the nucleus to the internal leaflet of the plasma membrane and because they are connected to the extracellular matrix via the integrins, they are components of an intrinsic system that regulate the mechanical properties of cells during cellular processes such as cell movement. Other prominent members of the intermediate filaments include the cytokeratins. Overexpression of cytokeratins such as KRT8 was shown to enhance adhesion of MCF7 cells to the extracellular matrix and correlate with drug resistance of breast cancer cells [33]. Godfroid *et. al.* further demonstrated the presence of cytokeratins KRT8, KRT18, and KRT19 on the outer surface of established human mammary carcinoma cells but not normal mammary cells[34]. In this study, up-regulation of Keratin 17 (KRT17) and down-regulation of Keratin 15 (KRT15) were observed in breast cancer. While there is little information on the role of KRT15 in breast cancer, KRT17 expression was studied in 600 breast tumors and was shown to be associated with poor clinical outcome [35].

#### (C) Focal and Cell adhesion and proteins

Assembly and disassembly of cytoskeletal proteins at focal adhesions are important steps during cellular movement. In line with the observation of aberrant expression of proteins associated with EMT and increased migratory potential, proteins such as Vinculin and Integrin A6 (ITGA6) that are involved in the regulation of focal adhesion were also found to be up-regulated. Furthermore, ITGA6 has been shown to be necessary for tumorigenesis of stem cell subpopulation derived from MCF7 cell line [36]. On the other hand, Galectin 3, a cell adhesion molecule, was detected to down-regulated in AT1K pre-neoplastic, CA1h low- and CA1a high-grade cancer cell lines compared to normal cells. This not surprising since cell-to-cell adhesion needs to be reduced in order for cancer cells to break apart and migrate. The ITRAQ data was confirmed by immunoblotting (Figure 3B) and is consistent with another study that described detectable expression of Galectin 3 in normal ducts and down-regulation in ductal carcinoma *in situ* [37].

#### (D) Others

Proteasomes are important for controlling the expressions of many proteins, one of the most prominent groups being the cell cycle regulating proteins. PSMB3 proteasome, which cleave peptides in an ATP/ubiquitin-dependent process in a non-lysosomal pathway, was found to be up-regulated in this study. This concurs with the observation that PSMB3 was co-expressed with ERBB2 in 34 breast cancer biopsies and also mapped within the same chromosomal location as the ERBB2 gene that is frequently amplified [38]. It is unclear how up-regulation of PSMB3 expression contributes to cancer. Valosin-containing protein is an ATPase that is involved in many cellular functions, including regulating the S26 proteasome function and E3 ubiquitin-protein ligase activity of RNF19A. High level of VCP expression in cancer cells has been shown to correlate with the increase in recurrence rate and poor prognosis of patients with cancer of the liver, stomach, prostate and esophagus [39]. A recent study identified VCP as an essential target of oncogenic Akt signaling and was necessary for cell growth and survival [40]. Up-regulation of VCP in at least two abnormal cell lines in MCF10AT model was confirmed by immunoblotting (Figure 3B).

### Aberrant expressions of novel-breast cancer associated proteins *in vitro* and ex-vivo

**Figure 4:**
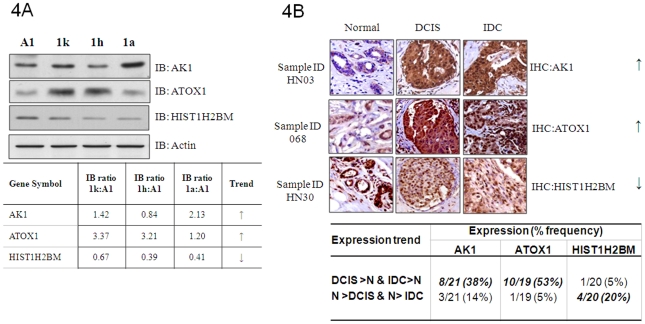
Validation of the expression of novel breast cancer-associated proteins AK1, ATOX1 and HIST1H2BM *in vitro* and *ex-vivo*. (**A**) **Top panels**, Immunoblotting of ATOX1, AK1 and HIST1H2BM. Cells were processed as per iTRAQ experiments. The lysates were immunoblotted with the protein-specific antibodies to reveal the relative expression levels across the 4 MCF10AT cell lines. Actin was included as a loading control. **Bottom panel**, Densitometry readings for the signals corresponding to the respective protein bands were obtained and expressed as a ratio using the signal from A1 normal cells as the denominator. (**B**) Immunohistochemistry of ATOX1, AK1 and HIST1H2BM were performed on 26 matched adjacent normal and tumor tissues containing a spectrum of lesions including DCIS and IDC. **Top panels**-A representative case showing the predominant expression trend for each candidate is shown. **Bottom panel**–Summary of the expression trends of candidate proteins between normal and breast cancer lesions.

From extensive literature search, AK1, ATOX1 and HIST1H2BM were among the few whose aberrant expressions have not been previously associated with breast cancer development. Therefore, we proceeded to conduct immunoblotting to examine the relative expression levels of these proteins in the MCF10AT model. The immunoblotting data and the densitometry values of the protein bands as well as the predominant expression trend of the proteins across the MCF10AT model are shown in Figure 4A, left panel. The proteins AK1 and ATOX1 showed up-regulation in one or more aberrant cell lines compared to A1 normal cells, while expression of HIST1H2BM was progressively decreased across the MCF10AT model. Although the absolute expression ratios generated by iTRAQ and immunoblotting methods are not the same, the expression trends reflected by both methods were congruent and confirmed the aberrant expressions of candidate proteins across the MCF10AT model of disease progression.

A major limitation of *in vitro* models like MCF10AT is that these systems lack the physiological context present in the human body. Consequently, we examined their expressions in 26 matched cases of clinical samples. From the immunohistochemistry studies, increased expression from matched normal to DCIS (ductal carcinoma *in situ*) and IDC (invasive ductal carcinoma) were observed for AK1 (8/21 or 38% of all cases) and ATOX1 (10/19 or 53% of all cases) (Figure 4B, lower panel). In contrast, decreased expression across progressive lesions was observed for HIST1H2BM in 20% (4/20) of all cases. Note that a significantly smaller frequency of opposite trend existed for most protein candidates, reflecting molecular heterogeneity of breast cancers. The raw IHC scores for the various candidate proteins are provided in Supplementary Table S4. One representative case of IHC data showing the predominant expression trend (up or down) for each candidate protein is shown in Figure 4B, upper panel. From the IHC data, the predominant localization of the candidate proteins could be summarized as: AK1–cytoplasmic and nuclear; ATOX1–cytoplasmic and nuclear; HIST1H2BM–nuclear. The observed localizations of these proteins are largely consistent with those reported in the literature or public databases such as GeneCards®.

## Discussion

This study represents the first proteome-wide approach to study the MCF10AT model of breast cancer progression. A DNA microarray study has been conducted recently on this model and some members in the S100 family were found to be down-regulated while Kallikrein and Thrombospondin were up-regulated [9]. In another study, array comparative genomic hybridization detected amplification of Myc and deletion of Runx 1, LRP1B, CDH13 and FHIT genes [10]. Due to the different nature of analytical methods used, these datasets largely do not overlap with ours, reiterating the complementary nature of proteomics and genomic approaches.

Using stable isotope/liquid chromatography-based mass spectrometry, we obtained the expression profiles of more than 1200 proteins in the MCF10AT model of breast cancer progression. The molecular changes detected suggest that cellular transformation and acquisition of aggressive phenotype involves “re-programming” of cellular systems, especially in processes associated with carbohydrate, amino acid and lipid metabolism. This is perhaps not surprising since metabolism is an Achilles' heel in cancer biology [41]; For instance, aberrant PI3K/AKT pathway during cancer development inadvertently amplifies glucose metabolism and translational activities via glucose transporter (GLUT4), mTOR/S6K/eukaryotic initiation factor 4E-binding protein 1 (4EBP-1), respectively [42], [43]. Consistently, several translation initiation factors were observed to be up-regulated in this study.

ATOX1, AK1 and HIST1H2BM have been validated as novel-breast cancer associated proteins. Among these, ATOX1 is most outstanding since its expression was higher in tumor compared to normal tissues in 53% (out of 19 cases) of matched clinical samples analyzed. ATOX1 was recently demonstrated to be a copper dependent transcriptional factor involved in cell proliferation [44]. Depletion of copper has been shown to inhibit angiogenesis in a variety of cancer and xenograft systems [45]. Several clinical trials using copper chelation as either an adjuvant or primary therapy have also been conducted [46]. It would be interesting to conduct further studies on ATOX1 that should provide new insights into the role and mechanism of ATOX1 in breast cancer biology.

The MCF10AT model is unlikely to reflect all the molecular changes associated with clinical breast cancers since it is an *in vitro* model. Besides, the isogenic cell lines in the MCF10AT model probably represent only one of the evolutionary tracks during tumorigenesis and do not encompass the entire spectrum of heterogeneity associated with breast cancers. Therefore, technical and/or biological factors therefore are likely to limit the resolution and the representativeness of the data presented in this study. Nevertheless, with these caveats in mind and appropriate validation steps taken, the mini-catalogue of protein expression changes should serve as a good reference/guide for future basic and translational cancer research. For example, not all cases of DCIS progress to carcinoma and whether all patients with DCIS should receive adjuvant therapy after breast-conserving surgery remains a topic of active debate [47]. Detection of the aberrant expression of ATOX1 and AK1 in pre-neoplastic cells (DCIS and AT1k cells) and the relatively higher expression of the two proteins in breast carcinoma compared to normal tissues suggest that they could be involved in cancer initiation and progression for at least a subset of breast cancers. It is conceivable that they could serve as molecular markers in determining the risk of DCIS developing local recurrence or invasive carcinoma and therefore help select patients for adjuvant therapy. On the other hand, proteins involved the later stages of cancer development might be important for disease maintenance/aggression and are potential prognostic factors and/or drug targets.

In conclusion, the information generated by this study should be a useful reference for future basic and translational cancer research. In turn, subsequent follow-up studies would eventually decipher which genes are cancer-driving and which are just passengers, thereby advancing our knowledge of breast cancer biology.

## Supporting Information

Table S1 Proteins with statistically significant expression changes across the MCF10AT model.
(0.06 MB XLS)

Table S2 Protein summary generated by ProteinPilot following mass spectrometry analysis.
(0.52 MB XLS)

Table S3 Classification of the proteins identified in the MCF10AT model into molecular functions using the PANTHER (http://www.pantherdb.org/).
(0.03 MB XLS)

Table S4 Raw IHC scores for candidate proteins in clinical breast samples.
(0.04 MB XLS)
